# A Review of Nanofiber Electrodes and the *In Situ* Exsolution of Nanoparticles for Solid Oxide Cells

**DOI:** 10.3390/ma18061272

**Published:** 2025-03-13

**Authors:** Jakub Lach, Michał Gogacz, Piotr Winiarz, Yihan Ling, Mingjiong Zhou, Kun Zheng

**Affiliations:** 1 Faculty of Energy and Fuels, AGH University of Krakow, al. A. Mickiewicza 30, 30-059 Krakow, Poland; jakublac@agh.edu.pl (J.L.); migogacz@agh.edu.pl (M.G.); pwiniarz@agh.edu.pl (P.W.); 2 School of Materials Science and Physics, China University of Mining and Technology, Xuzhou 221116, China; lyhyy@cumt.edu.cn; 3 School of Materials Science and Chemical Engineering, Ningbo University, Ningbo 315211, China; zhoumingjiong@nbu.edu.cn; 4 AGH Centre of Energy, AGH University of Krakow, ul. Czarnowiejska 36, 30-054 Krakow, Poland

**Keywords:** solid oxide cell, electrode material, nanofiber, in situ exsolution, nanoparticle, multi-elemental nanocatalyst

## Abstract

Solid oxide cells (SOCs) can operate efficiently in solid oxide fuel cell (SOFC) and/or solid oxide electrolysis cell (SOEC) modes, and are one of the most promising electrochemical devices for energy conversion and storage, facilitating the integration of renewable energies with the electric grid. However, the SOC electrodes suffer performance and stability issues, especially in the case of fuel electrodes when SOCs are fueled by cheaper and more available fuels such as methane and natural gas. Typical Ni-YSZ cermet fuel electrodes suffer problems of coarsening, carbon deposition, and sulfur poisoning. Therefore, developing new electrodes using novel design strategies for SOCs is crucial. In this review work, the fuel electrode development strategies including the in situ exsolution of nanoparticles, multi-elemental nanocatalysts, and nanofiber materials have been reviewed and summarized for the design of new electrodes for SOCs. Nanofiber electrodes with in situ exsolved nanoparticles, which combine the advantages of a unique nanofiber microstructure and stable and active exsolved nanoparticles, are of great interest and significantly contribute to the development of high-performance fuel electrodes for SOCs.

## 1. Introduction

For decades, the global demand for electricity energy has been growing rapidly, even exponentially [1]. According to the data, most of the energy supply comes mainly from coal or other fossil fuels, which are extremely unsustainable for society and the Earth [2]. For example, most of the nitrogen oxides released in the United States due to human activity are from the burning of fossil fuels associated with transportation and industry [3]. Air pollution causes severe diseases like heart disease, cancer, asthma, or even premature death [4]. Scientists still struggle to overcome this problem, searching for new sources of electrical energy that are clean, environmentally friendly, and safe for the human body. Hence, instead of traditional fossil fuels, renewable sources of energy are emphasized and are now an area of great interest. The greatest advantage is that they generate electricity without the contamination or pollution of the environment or atmosphere. Depending on the type of utilization, different kinds of energy sources are used. For example, in typical households, photovoltaic panels and small wind power plants are the most common. These devices generate electricity directly from solar and wind energy. On the other hand, sources of energy that are independent of the weather or other environmental aspects are in high demand. One of the most promising sources of energy which combines high effectiveness, durability—while simultaneously giving high power densities—is the fuel cell. This electrochemical device produces electricity through simple electrochemical reactions, without any combustion processes. A typical fuel cell consists of three components: an anode, cathode, and the electrolyte layer between them. From the chemical point of view, in regenerative mode (electrolysis), the anode plays the cathode role; therefore, the term “air electrode” (instead of slightly misleading anode/cathode) is more precise for solid oxide cells. Moreover, SOFCs can be merged to form a stack, providing extraordinary power densities, in terms of megawatts. For instance, an American company, BloomEnergy, provides SOFCs stacks to other customers. They installed a 1 MW fuel cell for the Honda company, which enabled a significant reduction in the unwanted production of CO_2_ at this company [5]. All in all, in the field of new clean power sources, SOFCs are one of the most trending objects of interest, and are still being improved to achieve the highest power densities with the smallest financial outlay.

## 2. Solid Oxide Fuel Cells and Solid Oxide Electrolysis Cells

**Figure 1:**
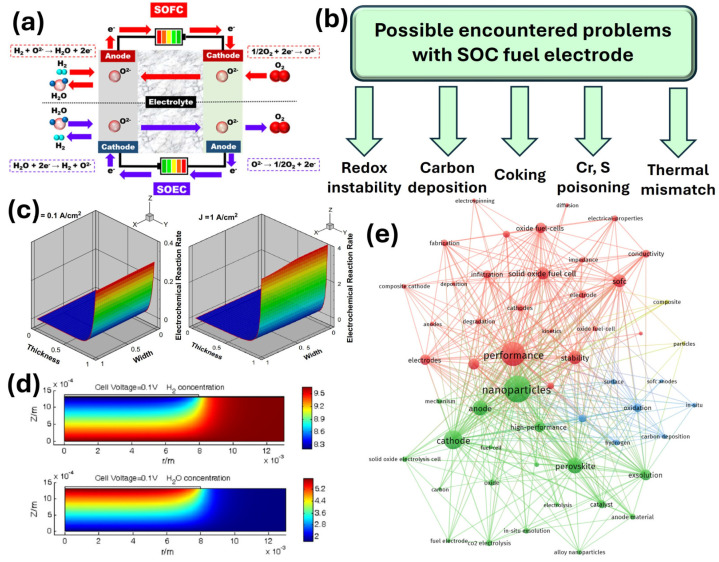
(**a**) Schematic of SOFC/SOEC operation. Reproduced with permission [7]. Copyright 2020, Wiley. (**b**) Problems related to the SOC fuel electrode side caused by different phenomena. (**c**) Distribution of hydrogen oxidation reaction rate within the anode at different current densities at 800 °C. Reproduced with permission [14]. Copyright 2009, Elsevier. (**d**) Numerical distributions of the H_2_ and H_2_O concentrations at the fuel electrode. Reproduced with permission [16]. Copyright 2007, Elsevier. (**e**) Search methodology diagram of the literature terms and their connections utilized in this review. Web of science keyword co-occurrence mapping knowledge domain.

There are many different kinds of fuel cells depending on the working temperature, type of fuel, or electrolyte, e.g., [6], molten-carbonate fuel cells operate roughly at 600–700 °C and require either biogas or carbon monoxide/dioxide as a fuel. However, a lot of disadvantages, such as the fast corrosion of components, low power densities, and a long turning on time, make it almost impossible for commercial usage. On the other hand, alkaline fuel cells operate at temperatures around 200 °C using hydrogen as fuel, where electrodes are saturated with an aqueous alkaline solution. Their major drawbacks are that they are liquid electrolytes and are not CO_2_-resistant. Therefore, solid oxide fuel cells (SOFC) appear to be the most promising energy conversion and storage technology. They possess the most advantages, i.e., the possibility of usage in many applications, the possibility of applying different fuels, and the possibility of using different catalysts. Their operation, along with electrochemical reactions occurring at the air electrode side (cathode in SOFC and anode in SOEC) and fuel electrode side (anode in SOFC and cathode in SOEC) is presented in Figure 1a [7]. The open circuit voltage (OCV) produced through electrochemical reactions, according to the Nernst equation, is shown in Equation (1):

(1) $$OCV=\frac{RT}{4F}ln\left(\frac{pO_{2\left(cathode\right)}}{pO_{2\left(anode\right)}}\right)$$

where R is the gas constant approximately equal to 8.31 J·mol^−1^·K^−1^, T is absolute temperature, F is Faraday constant approximately equal to 96,485 C·mol^−1^, and the instances of pO_2_ are the respective values of oxygen partial pressure at the cathode and anode side. As an example, at a given constant temperature of 500 °C (773.15 K), when a SOFC is fed by pure oxygen and hydrogen, the OCV is around 1.08 V.

The ongoing energy transition towards green and sustainable sources aims to achieve significant reductions in greenhouse gas emissions by 2030–2050 [8]. Hydrogen is seen as a key energy carrier due to its ability to store, transport, and generate green energy. However, fossil fuels remain the dominant source of hydrogen production, typically through methods like steam reforming, coal gasification, or the partial oxidation of heavy hydrocarbons. On the other hand, water electrolysis offers a cleaner alternative for hydrogen production. This process involves splitting water molecules into hydrogen and oxygen using electrical current. According to the literature, this operation mode is also called SOFC regenerative mode, which is used to achieve the electrolysis of water; therefore, these electrochemical devices are named solid oxide electrolysis cells (SOECs). SOECs have undergone significant development over the past years. Operating at elevated temperatures (typically 600–800 °C), SOEC technology can substantially reduce the amount of power required to split water into hydrogen, which in turn enhances power-to-hydrogen efficiency.

When the advantages of solid oxide fuel cells and solid oxide electrolysis cells are merged, they are often called reversible solid oxide cells, or, in short, solid oxide cells, which is a more widely used term. Under external electrical voltage, a solid oxide electrolysis cell (SOEC) separates H_2_O into hydrogen by transporting oxygen ions through a conductive membrane that after recombination with electrons forms oxygen molecules. Since the excess heat is integrated into the SOEC process, the efficiency of SOEC systems can be higher than other electrolysis technologies. SOECs work at temperatures higher than 700 °C and lower than 1000 °C, enabling nonprecious metals to act as catalysts. Therefore, one of the main advantages of water electrolysis in SOEC the production of pure H_2_ from the steam without any combustion processes. Solid oxide electrolysis cells also exhibit a great advantage in the direct production of H_2_ and CO, which is called “syngas”, through the electrolysis process which simultaneously converts H_2_O and CO_2_ [9].

In general, the majority of SOFCs are fed by air on the air electrode side and by hydrogen-rich fuel on the fuel electrode side to achieve a high OCV. Hydrocarbons such as methane (CH_4_) or other gases such as ammonia (NH_3_) are also used; however, one can expect problems to be encountered on the fuel side, e.g., carbon deposition [10], coking [11], chromium poisoning [12] or thermal mismatch [13] caused by thermal stresses. These are schematically shown in Figure 1b.

Numerical calculations, modeling, or simulations such as computational fluid dynamics (CFD) are complementary methods that allow an understanding of the phenomena that occur in the whole SOC or similar electrochemical devices on the fuel electrode/electrolyte or air electrode/electrolyte interfaces. According to Hussain et al. [14], the anodic reaction rate may be tested at different current densities and different temperatures, which is schematically shown in Figure 1c. In effect, interesting conclusions may be drawn, i.e., the part of the fuel electrode exhibits some changes in the electronic current and ionic current, which indicates that the reactive sites where the electrochemical reaction is most active can be considered as the electrode reaction zone layer. What is more, the reaction zone layer thickness increases with the increase in the operating temperature. Also, some theoretical calculations or tests are also needed to check if the assumptions meet the requirements. As shown by Dong et al. [15], coupled electrochemical reactions allow the prediction of some transport processes, e.g., mass, momentum, and heat transfers, enabling the use of the data prior to design and further synthesis. What is more, Shi et al. presented numerical calculations of hydrogen and water distribution on the fuel electrode side [16], which is shown in Figure 1d. The model was applied to generate polarization curves along with parameter distributions for different cell configurations using the given operating conditions. The obtained results suggest that the electrochemical reactions occur mainly in the vicinity of the electrode/electrolyte interface, and with decreasing hydrogen content in the fuel, the reaction occurs at a position further from the anode/electrolyte interface.

To understand and overcome the aforementioned problems, different improvements and enhancements are made. For instance, these electrochemical processes may be diminished by adjusting the porosity of the anode and operating current density in conventional Ni anodes [17] and by adjusting the gas concentration in feeding gas [18]. However, recently, new ideas have emerged for finding solutions to these issues, which are described in the next chapter. Since the research focusing on improving the performance of solid oxide cells is still ongoing and many ideas are being implemented to achieve this goal, a comprehensive diagram showing the connections between the literature terms is presented in Figure 1e, highlighting the search methodology utilized in this review. As can be seen from the diagram, the exsolution process or electrospinning method is still not extensively investigated and discussed; therefore, it is extensively described in this article.

## 3. Fuel Electrodes Design for Solid Oxide Cells

From the materials point of view, one of the most common types of fuel electrodes is composed of ceramic and metal (cermet), these being yttria-stabilized zirconia (YSZ) and nickel, respectively. This material combines the most desirable features, i.e., low cost, good chemical stability in reducing atmospheres and in high temperatures (up to 1000 °C) [19], and a similar thermal expansion coefficient to the YSZ electrolyte [20]. Generally, the majority of problems connected with fuel electrodes are connected with drawbacks presented by Ni, e.g., it is not resistant to re-oxidation, carbon incorporation (coking) in carbon-rich fuels, and deactivation by fuel contaminants (e.g., sulfur) [21]. Properly designed fuel electrodes should exhibit a TEC (thermal expansion coefficient) similar to the chosen electrolyte material to prevent thermal mismatch which causes cell degradation, cracks, and additional buffer layer delamination. Since the electrolyte material should exhibit high thermally activated oxygen ion conductivity, oxide materials with large amounts of oxygen vacancies are used, e.g., YSZ [22], ceria-based materials like Ce_0.9_Gd_0.1_O_2−δ_ (GDC) [23], or La_0.9_Sr_0.1_Ga_0.8_Mg_0.2_O_3−δ_ (LSGM) [24]. Therefore, fuel electrodes should also be thermomechanically and chemically compatible with these electrolytes, which can be experimentally checked by mixing powders, heating them together, and examining by means of X-ray diffraction, which is as presented in [25].

**Figure 2:**
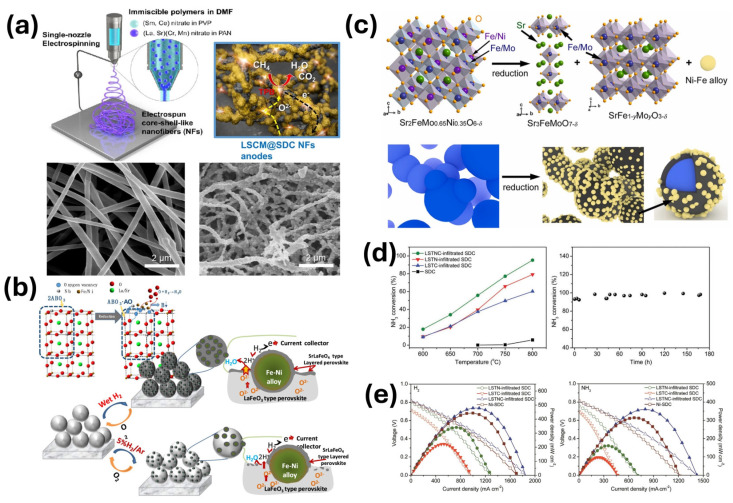
(**a**) Robust and highly active core–shell La_0.75_Sr_0.25_Cr_0.5_Mn_0.5_O_3_@Sm_0.2_Ce_0.8_O_1.9_ anodes with a nanofibrous structure, which were obtained directly using a single-nozzle electrospinning process with two immiscible polymers. Reprinted with permission from [29]. Copyright 2022, American Chemical Society. (**b**) Multifunctional nanoarchitectured La_0.5_Sr_0.5_Fe_0.8_Ni_0.1_Nb_0.1_O_3−δ_ anode with improved properties obtained as a result of the exsolution process. Reprinted with permission from [33]. Copyright 2020 American Chemical Society. (**c**) Structural transition in Sr_2_FeMo_0.65_Ni_0.35_O_6−δ_ giving Ni-Fe exsolved nanoparticles. Reprinted with permission [34]. Copyright 2024, Elsevier. (**d**) Ammonia conversion rate in different fuel electrodes (**left**), ammonia conversion stability as a function of time (**right**). Reprinted with permission from [35]. Copyright 2020. Wiley. (**e**) Peak power densities for La_0.52_Sr_0.28_Ti_0.94_Ni_0.03_Co_0.03_O_3−δ_ (LSTNC) operating in H_2_ (**left**) and NH_3_ (**right**) as fuel. Reprinted with permission from [35]. Copyright 2020. Wiley.

As mentioned in Figure 1b, typical Ni-YSZ cermets may exhibit redox instability and are not resistant to methane (CH_4_) or sulfur poisoning, therefore, different perovskites and double perovskites are still being investigated as good fuel electrode materials with sufficient electrochemical properties, such as A-site deficient (La_0.3_Sr_0.7_)_1−x_TiO_3−δ_ [26], Sr_2_Mg_1−x_Mn_x_MoO_6−δ_ [27], or La_0.75_Sr_0.25_Cr_0.5_Mn_0.5_O_3−δ_ [28]. One of the novel ideas is to obtain porous fuel electrodes with the electrospinning method. According to Choi et al. [29], nanofibrous core–shell La_0.75_Sr_0.25_Cr_0.5_Mn_0.5_O_3_@Sm_0.2_Ce_0.8_O_1.9_ fuel electrodes can be obtained which exhibit lower polarization resistance when compared to similar materials. The schematic of electrospun materials, with the respective scanning electron microscope (SEM) images, is shown in Figure 2a.

Nevertheless, recently, many reports regarding the exsolution process emerged which allow the enhancement of the electrochemical properties of fuel electrodes; therefore, such materials are, again, gaining attention [30,31,32]. In single perovskites, the process may be described as follows:

(2) $$2ABO_{3}\left(surface\right)\overset{reducing\;conditions}{\to }\left(ABO_{3}\cdot AO\right)+B↓+2O\left(V_{O}^{\cdot \cdot }\right)$$

As reported by Wu et al., multifunctional architectured core–shell nanostructures of La_0.5_Sr_0.5_Fe_0.8_Ni_0.1_Nb_0.1_O_3−δ_ (LSFNNb0.1) were constructed using a one-step process [33], which is schematically shown in Figure 2b. In the single cell composed of LSFNNb0.1|SDC|ScSZ|SDC|LSFCN, the authors recorded a maximum power density equal to 830 mW·cm^−2^ at 800 °C in wet H_2_. A similar approach was presented by Xia et al. in Sr_2_FeMo_0.65_Ni_0.35_O_6−δ_, where under reducing conditions, Ni-Fe nanoparticles were obtained, giving enhanced electrochemical properties [34]; this is presented in Figure 2c. Perovskite anodes also work efficiently using ammonia as a fuel. A direct ammonia solid oxide fuel cell (DA-SOFC) operating in low temperatures was reported by Song et al. [35].

## 4. Nanofibers with In Situ Exsolution of Nanoparticles

### 4.1. In Situ Exsolution of Nanoparticles

**Figure 3:**
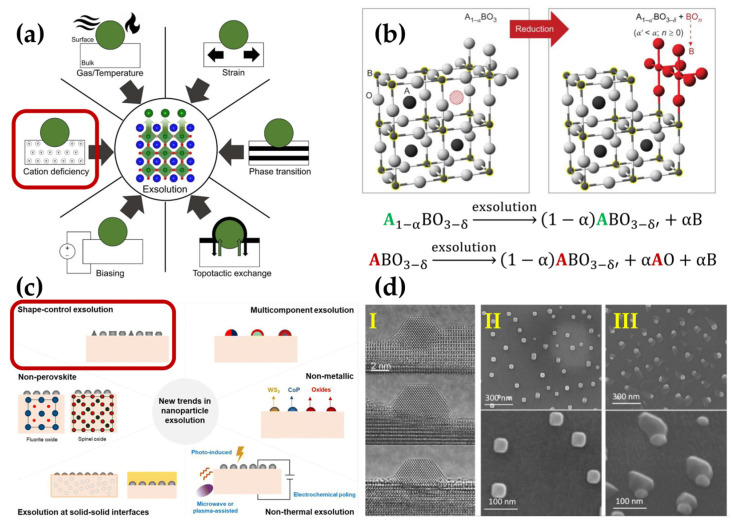
(**a**) Strategies used to tune the exsolution process from perovskite oxides: operating conditions, oxide nonstoichiometry, electrical voltage, topotactic reaction, phase transition, and compressive or tensile strain. Green spheres represent exsolved nanoparticles. In the depicted crystal structure, blue, green, and red spheres represent A-site ions, B-site ions, and oxygen anions, respectively. Reprinted with permission from [38]. Copyright 2020, IOP Publishing. (**b**) Schematic illustration of the exsolution of B-site ions from the nonstoichiometric perovskites of the A-site. The large silver spheres represent O-site ions, small gray spheres accentuated in yellow represent B-site ions, and large dark spheres represent A-site ions. The large red hatched sphere indicates an A-site vacancy. Reaction equations compare the process of exsolution from perovskites with A-site deficiency and cation stoichiometric perovskites. Reprinted with permission from [40]. Copyright 2013, Springer Nature. (**c**) The six new trends in nanoparticle exsolution. Reprinted with permission from [41]. Copyright 2024, Royal Society of Chemistry. (**d**) Advanced exsolution process based on nanoparticle shape: (**I**) faceted particles grown from La_0.43_Ca_0.37_Ti_0.94_Ni_0.06_O_3_ perovskite under vacuum in environmental transmission electron microscopy at 900 °C. (**II**) Cubic-shaped particles grown from La_0.8_Ce_0.1_Ti_0.6_Ni_0.4_O_3_ in a 5% CO environment at 900 °C. (**III**) Ellipsoid-shaped particles connected with as-grown oxide (La_2_TiO_5_) under wet 5% H_2_ atmosphere from La_0.8_Ce_0.1_Ti_0.6_Ni_0.4_O_3_ perovskite at 1000 °C. Reprinted with permission from [42]. Copyright 2019, American Chemical Society. The issues surrounded by red squares in subfigures (**a**) and (**c**) are expanded in subfigures (**b**) and (**d**), respectively.

Typically, the A-site cations are composed of lanthanides, such as La, Nd, Sm, or alkaline earth metals, like Ca, Sr, and Ba. Meanwhile, the B-site cations are composed of smaller transition metals, such as Mn, Fe, Co, and Ni. The catalytic properties of perovskite oxides (ABO_3_) are greatly influenced by the coordination of A- and B-site cations [36]. The choice of A-site cations (lanthanides) significantly affects the crystal structure (simple ABO_3_ or double AA’BB’O_6_ perovskite structure), while the stability of the materials in air and reducing atmospheres is mainly determined by the B-site cations. Under reducing conditions and SOC operating temperatures, the perovskite oxides can undergo a partial decomposition process. This process is called exsolution, where B cations move out of the bulk and are reduced to their metallic state, leading to nucleation and particle growth and the formation of nanocatalysts [36,37]. Gao et al. suggested that exsolution arises from the four physical processes of diffusion, reduction, nucleation, and growth [37]. During the exsolution, many factors govern this process, such as temperature, cation nonstoichiometry, voltage biasing, strain, phase transition, and topotactic ion exchange (see Figure 3a). All of these factors directly influence the process of oxygen vacancy formation, which is referred to as the driving force for the exsolution process [38].

To predict which ions can be selectively exsolved, it is important to consider the reducibility of individual ions or corresponding oxides. In this way, it can be understood which ions could be exsolved to form nanoparticles and which ones remain in the parent lattice in ionized form [36]. By comparing the ΔG (Gibbs free energy change) of the reduction of different metal oxides, it is evident that practically all A-site ions are not reducible to the zero-valent state under typical SOCs anode conditions (reducing atmosphere, 1000 K). Only certain elements from the B sublattice (such as Pd, Fe, Co, Ni, and Cu) are capable of being exsolved onto the oxide surface and reduced to the metallic state, while other metals (such as Mn) will remain in an ionic state within the perovskite lattice under these conditions [36].

During the design of materials covered by exsolved nanoparticles, the introduction of A-site nonstoichiometry into the perovskite structure is regarded as highly beneficial. A-site deficiency can significantly improve the catalytic activity of the electrode as a result of the formation of additional oxygen vacancies, which are critical for the transport of oxygen ions in the electrode [39]. Furthermore, this strategy facilitates the self-generation of nanocatalysts on the surface of perovskite oxides under reducing conditions, as shown in Figure 3b. Considering the reaction equations shown in Figure 3b, it can be stated that perovskite oxides with A-site cation nonstoichiometry are better suited for the exsolution of metallic nanoparticles than those without A-site cation deficiency [40].

It is worth noting that the exsolution process is not limited to metallic nanocatalysts exsolved from perovskite oxides. Research is being conducted on compounds where the parent oxides have a fluorite or spinel structure. Moreover, the nanoparticles can be nonmetallic and be exsolved at solid–solid interfaces, not only at elevated temperatures, but also under an applied electrochemical poling, as shown in Figure 3c [41]. In addition to the modifications in the size and density of exsolved nanoparticles, the shape is also particularly important, as it can determine the electrochemical properties of nanocatalysts [42]. For example, it was found that the exposed surface area of polyhedral-shaped nanoparticles is larger than that of spherical ones for the same volume [41]. One of the parameters determining the shape of the exsolved nanoparticles may be pressure and the atmosphere of the surrounding gas during the exsolution process, as shown in Figure 3d [42].

**Figure 4:**
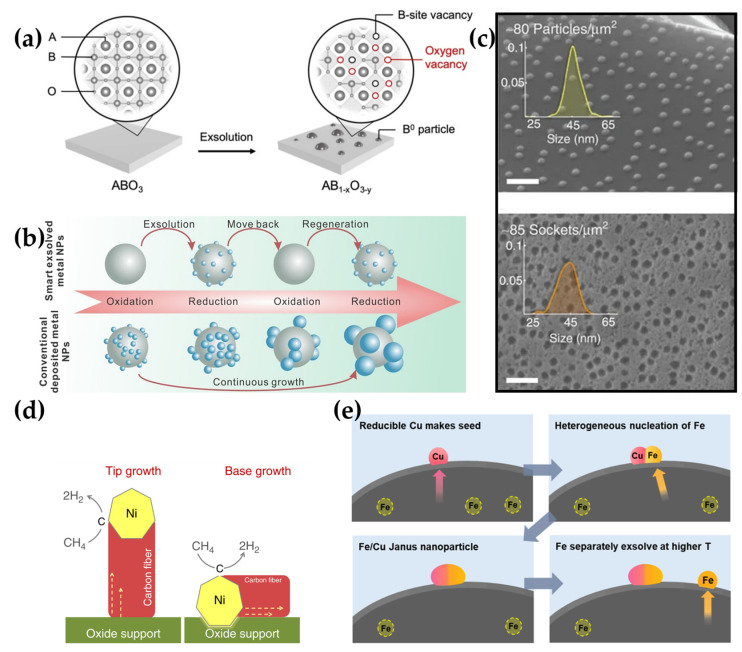
(**a**) Schematics illustrating the exsolution process from perovskite (ABO_3_) oxides. Besides surface modification, the exsolution process influences the bulk properties of the electrode material by increasing the concentration of oxygen vacancies concentration within the perovskite structure. Reprinted with permission from [46]. Copyright 2023, IOP Publishing. (**b**) Diagram with a comparison of nanocatalysts properties, obtained with the exsolution and conventional deposition processes. Reprinted with permission from [52]. Copyright 2020, American Chemical Society. (**c**) SEM micrographs of exsolved Ni particles from La_0.52_Sr_0.28_Ni_0.06_Ti_0.94_O_3_, after reducing in 5% H_2_ in argon at 920 °C for 12 h, before and after etching particles in HNO_3_, with size histograms of the exsolved particles and sockets, respectively. SEM micrographs indicate an anchoring mechanism of exsolved nanoparticles. (**d**) Schematic of possible carbon fiber growth mechanisms in the material with weakly embedded and well-anchored Ni nanoparticles in oxide support, respectively. Reprinted with permission from [53]. Copyright 2015, Springer Nature. (**e**) Schematic illustration of how the exsolution process occurs, according to the seeded effect mechanism. Reprinted with permission from [58]. Copyright 2025, Elsevier.

To boost performance and improve stability, designing materials capable of the exsolution of nanocatalysts is considered highly beneficial for use in SOC electrodes [43,44,45]. Since exsolution occurs under a reducing atmosphere, a primary driving force for exsolution is the formation of oxygen vacancies in the perovskite structure. The increased concentration of oxygen vacancies destabilizes the lattice stoichiometry, leading to the segregation and exsolution of B-site cations to maintain the host crystal structure (see Figure 4a). In this way, exsolution is also considered a partial decomposition process and has an impact not only on surface properties but also on the bulk modification of electrode material [38,46,47]. The increasing concentration of oxygen vacancies within the perovskite structure may have a positive impact on ionic conductivity. In perovskite oxides, the ionic part of conductivity is realized by the oxygen vacancy mechanism. The generation of oxygen vacancies can be particularly advantageous for electrode materials because it creates fast channels for oxygen ion diffusion (charge carriers), promoting an increased ionic conductivity [45].

The exceptional results in the literature indicate that exsolved metallic nanoparticles enhance catalytic activities and stability, also leading to the improved performance of SOCs in lower temperature ranges (below 600 °C). The low-temperature operation of reversible SOCs (r-SOCs) can effectively improve their stable and economical application. In the anode operation condition, metallic nanoparticles can be exsolved from the parent oxides decorating the electrode surface, substantially improving the catalytic activity and electronic conductivity of the anode. Furthermore, partially submerged or socketed nanoparticles in the perovskite oxide matrix improve the metal-support interaction, retarding coalescence sintering and inhibiting the growth of the exsolved nanocatalysts. The nanoparticles can be moved in and out of the perovskite matrix under oxidation and reducing conditions [36,44,48,49,50,51]. Therefore, exsolution is a promising alternative to conventional impregnation methods to obtain robust nanocatalysts on the electrode surface [41]. The conventional wet impregnation involves mixing solutions of the desired metals (typically metal salts, such as nickel nitrates) with ceramic oxide support, allowing water absorption to carefully cover the material’s surface with the added metal. The next step involves water evaporation and calcination. However, due to poor size control, nonuniform distribution, and weak interaction with the parent material, using this method is limited for SOCs electrodes [51,52]. Compared to exsolved nanocatalysts, the infiltrated nanoparticles exhibit a tendency to grow and sinter, especially at higher temperatures, as shown in Figure 4b [52]. Vera et al. compared exsolved and impregnated Ni nanoparticles on La_0.52_Sr_0.28_Ti_0.94_Ni_0.06_O_3_ perovskite for low-temperature CO oxidation and confirmed that both methods providing nanoparticles allow similar catalytic activity and stability at low-temperature carbon oxide (II) oxidation. However, after an aging process at 800 °C in an oxidizing atmosphere, the initial catalytic activity of impregnated nanoparticles was 10 times lower than of exsolved nanocatalysts, which authors attributed to probable sintering and the growth of Ni nanoparticles, as well as the weak interaction with the parent oxide [51]. The socketed to the perovskite in situ exsolved nanocatalysts have a lower tendency for particle growth, enhancing long term stability and hydrocarbon coking tolerance [38]. In Figure 4c, the morphology of La_0.52_Sr_0.28_Ni_0.06_Ti_0.94_O_3_ perovskite is shown, both before and after (top and bottom, respectively) etching in concentrated HNO_3_. The similar size distribution of exsolved nanoparticles and pits after etching confirms the socketing mechanism of exsolved Ni nanoparticles [53].

Moreover, the nanoparticles can be moved out and in of the perovskite matrix under oxidation and reducing conditions, respectively [47,51]. This reversible exsolution/dissolution of nanoparticles can potentially resolve the tendency to particle agglomeration during long-term operation and coke formation while SOC is fueled by non-hydrogen fuels [47,53]. Due to its intriguing behavior, the reversible exsolution process has attracted significant research attention, leading to many measurements in this field. Lv et al., using in situ measurements, found that CoFe nanoparticles, exsolved from Sr_2_Fe_1.35_Mo_0.45_Co_0.2_O_6–δ_ perovskite, are oxidized to flat CoFeO_x_ (likely CoFe_2_O_4_ spinel) nanoxides at 600 °C, and then completely dissolved in parent perovskite at 800 °C. Nanoparticles undergo oxidation before dissolution, which may result from the more energy-intensive process of dissolution into the host oxide than alloy oxidation. Interestingly, during the dissolution of nanoparticles, a phase transition of parent oxide was found from layered perovskite to double perovskite. The reverse phase transition was observed during the metallic nanoparticles exsolution in a reducing atmosphere [47]. During carbon poisoning, carbon may be dissolved into metallic nanoparticles (such as Ni), causing fiber growth at the metal–oxide interface. This process lifts the nanocatalysts from their previous position, and is observed especially for Ni particles, due to its high activity towards carbon deposition in hydrocarbon fuels. Therefore, many efforts are made to replace infiltrated Ni with other metals (such as Cu and Pd) for hydrocarbon utilization [51,53,54]. The anchored mechanism in exsolved nanoparticles can reduce this fiber growth due to the strong interaction between metallic particles and oxide support, which improves carbon coking resistance and enables the SOCs to be fueled by cheaper and more available non-hydrogen alternatives (see Figure 4d) [53].

It is worth adding that the exsolution process is not always reversible. Exsolved nanoparticles cannot return to the perovskite structure in an oxidizing atmosphere, as was found in La_0.8_Ce_0.1_Ni_0.4_Ti_0.6_O_3_ and La_0.7_Ce_0.1_Co_0.3_Ni_0.1_Ti_0.6_O_3_ materials. The Ni and Co-Ni metallic nanocatalysts do not re-dissolve back into the perovskite host lattice [38,55]. Zhang et al. designed a novel Sm_0.9_Ba_0.9_Mn_1.8_Co_0.2_O_5+δ_ electrode for symmetrical SOFCs. They found that in operating conditions, the perovskite is co-decorated with exsolved cobalt nanoparticles for the anode and cobalt oxide for the oxygen electrode layer. Impedance spectroscopy with the distribution of relaxation times analysis confirmed that Co and Co_3_O_4_ nanocatalysts significantly promote the adsorbed hydrogen dissociation process at the anode and charge transfer process at the cathode, boosting the efficiency of SOFC [45].

Certain transition metal ions can be induced to be exsolved from perovskite by surface modification processes, such as topotactic ionic exchange and the seeded effect. Topotactic exsolution involves exchanging host cations and the guest cations deposited on the material surface. The difference in segregation energy between the host and guest cation is regarded as a crucial driving force in topotactic ionic exchange [47]. Zhang et al. triggered the Ni exsolution and tuned the nanoparticle density on Pr_0.7_Sr_0.3_Cr_0.9_Ni_0.1_O_3−δ_ perovskite infiltrated with Fe as a result of topotactic exsolution, where Fe ions act as the guest cations. This led to obtaining reduced and stoichiometric Pr_0.7_Sr_0.3_Cr_0.9_(FeNi)_0.1_O_3−δ_ perovskite, covered by FeNi alloy nanoparticles. The density of FeNi nanoparticles increases with the increase in iron amount. The recorded maximum electrochemical performance of 1.49 A cm^−2^ at 800 °C and 1.6 V for the SOEC cathode for CO_2_ electrolysis demonstrated that the topotactic ion-exchanged cathode improved by over 62.0% compared to the perovskite without Fe infiltration. Interestingly, no exsolution phenomena were observed on the reduced Pr_0.7_Sr_0.3_Cr_0.9_Ni_0.1_O_3−δ_ oxide without Fe addition on the surface [56]. The seeded effect is also considered as an efficient method to trigger nanocatalyst exsolution and is based on triggering the exsolution of doped cations, without ionic exchange between host and guests’ cations (see Figure 4e). Compared with the topotactic exsolution process, in this case, ionic exchange between the host and guest does not occur [57,58]. The process principles can be described on La_0.43_Sr_0.37_Fe_0.09_Cu_0.03_Ti_0.88_O_3−δ_ perovskite. Jo et al. found that Cu is first exsolved from the perovskite on the oxide surface and plays a key role in triggering the exsolution of Fe from the B-site sublattice. The Cu particles stimulate the iron exsolution from perovskite, forming Janus heterostructure nanocatalysts that exhibit high catalytic activity for H_2_O and CO_2_ electrolysis in r-SOCs. The remaining Fe cations in perovskite can be exsolved independently at higher temperatures [58].

Due to the intriguing properties of materials coated with metallic nanocatalysts, advanced research utilizing advanced methods such as synchrotron radiation is being conducted to elucidate the in situ exsolution process. Najimu et al., in order to clarify the reducing process, performed spectroscopic experiments, which involve in situ X-ray absorption spectroscopy (XAS) and absorption near-edge structure (XANES) spectroscopy in 5 vol% H_2_ in He atmosphere, and found that in the LaFe_0.8_Ni_0.2_O_3_ compound, the transition of Ni^3+^ (from perovskite lattice) to metallic Ni^0^ starts at ~270 °C with the formation of Ni nanoparticles, while the transition of Fe^3+^ to Fe^0^ with the formation solid solution with Ni occurs at ~700 °C and forms Ni-Fe nanoparticles [48]. Steiger et al. have used XANES and X-ray fluorescence spectroscopy (XRF) to perform the ex situ measurements of reversible exsolution and the dissolution of Ni nanoparticles from La_0.3_Sr_0.55_Ti_0.95_Ni_0.05_O_3−δ_ perovskite oxide. The obtained results allow them to determine the oxidation state of nickel and distinguish bond types of Ni with neighborhood elements [59]. Napolitano et al. have used XANES to determine the oxidation state of Co and Ti cations in La_0.4_Sr_0.6_Ti_1−y_Co_y_O_3±d_ perovskite based on the obtained values of calculate oxygen deficiency in perovskite [60]. Also, in situ measurements of the exsolution process can be successfully performed, as shown by the example of Sr_0.93_Ti_0.3_Fe_0.63_Ni_0.07_O_3_ and Sr_0.93_Ti_0.3_Fe_0.56_Ni_0.07_Co_0.07_O_3_ perovskites. The use of two material reduction methods allowed the authors of one study to explain the exsolution mechanism and determine the temperature range of the process [61].

### 4.2. Multi-Elemental Nanocatalysts

Alloys are one of the most popular substances with tunable properties, where atoms are randomly distributed or packed in an ordered manner and exhibit advantageous catalytic properties arising from interactions between multiple elements [62]. Multicomponent alloys, due to their outstanding properties, have attracted wide interest as catalytic materials over the last few years. According to the nomenclature, alloys containing five or more elements with similar atomic rations are classified as high-entropy alloys (HEAs) and are considered as a rising star family of robust materials for catalysis. The phase stability of multicomponent alloys can be estimated with the use of the Gibbs free energy equation:

(3) $${\Delta G}_{mix}={\Delta H}_{mix}-{T\Delta S}_{mix}$$

where ${\Delta G}_{mix}$, ${\Delta H}_{mix}$, ${\Delta S}_{mix}$, and T are the changes in the Gibbs free energy, mixing enthalpy, mixing entropy, and temperature, respectively. When ${\Delta G}_{mix}<0$, single-phase with random distribution is favored, while ${\Delta G}_{mix}>0$ indicates a thermodynamic-driven separation of the multicomponent phase. From a configurational point of view, a more disordered system and higher randomness of structure with a lower Gibbs free energy (ΔG = H − TΔS) can facilitate the stability of the host structure, especially during catalysis at higher temperatures [63]. The mixing entropy can be expressed according to the equation below [62]:

(4) $${\Delta S}_{mix}=-R\sum x_{i}ln\;x_{i}$$

where R represents the gas constant and $x_{i}$ denotes the mole fraction of the selected metal.

**Figure 5:**
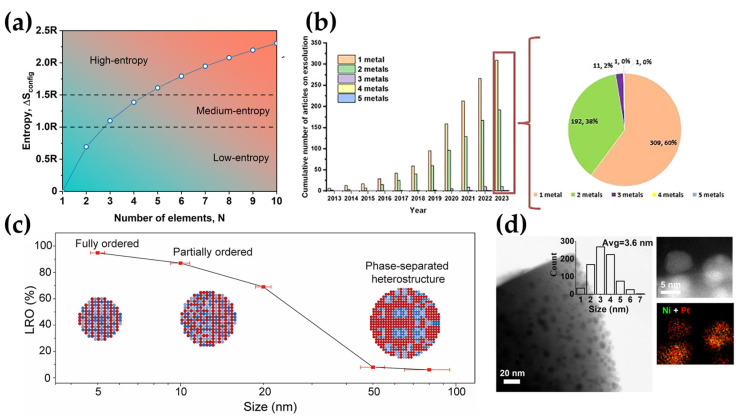
(**a**) The ideal configurational entropy depending on the number of equiatomic elements. Reprinted with permission from [71]. Copyright 2025, Elsevier. (**b**) Number of articles published in selected years on exsolution of one to five metals. Reprinted with permission from [48]. Copyright 2022, The American Association for the Advancement of Science. (**c**) The long-range ordering (LRO) as a function of the quinary multi-principal element intermetallic compound grain size, with insets schematically showing the microstructural evolution. Reprinted with permission from [72]. Copyright 2022, The American Association for the Advancement of Science. (**d**) Scanning transmission electron microscopy (STEM) image and energy dispersive X-ray spectroscopy elemental map of the Ni-Pt nanoparticles for La_0.52_Ca_0.28_Ni_0.06_Ti_0.94_O_3_-Pt nanofibers reduced at 800 °C in 5% H_2_/Ar, with a size distribution of nanocatalysts. Reprinted with permission from [73]. Copyright 2024, Springer Nature.

It can be calculated that the equimolar fraction of composition maximizes the value of ${\Delta S}_{mix}$. Moreover, with the rising number of alloys, the changes in mixing entropy increase from 1.10R to 1.61R for equiatomic ternary and quinary alloys, respectively. In light of the above, it can be concluded that configurational entropy can be a key factor in ensuring the phase stability of a multicomponent systems, especially at elevated temperatures. Moreover, when multicomponent systems consist of more elements with near-equal mole ratios, this significantly increases the entropic contributions, which consequently decreases the overall Gibbs free energy (see Figure 5a) [62]. In HEAs, different elements due to the interaction form unexpected surface electronic structures, may create a diverse array of continuous catalytic sites, which is known as the cocktail effect [64]. The reaction mechanism of the catalytic process is fundamentally influenced by entropy-driven behaviors of molecular species, including translation, rotation, and the vibration of intermediates bound to active sites, as well as the adsorption of reactants. Therefore, HEAs have been comprehensively studied [62].

These characteristics of HEAs lead to some advantages over the conventional bi- and three-element alloys, such as in their corrosion resistance, along with their outstanding physiochemical and surface properties, including exceptional catalytic activity and the ability to self-regenerate during catalytic redox processes. Simultaneously, higher diffusion activation energies and sluggish diffusion kinetics in HEAs enhance chemical, thermal, and mechanical stability [62,63,65,66]. Various effects in high-entropy alloys, such as the strain effect, ligand effect (spatial distribution of different elements), and coordination effect (alloy geometries) tune alloy surface catalytic activity, and play beneficial roles in the catalytic properties [67]. Lattice distortion generated in the crystal structures by the atomic radius difference inhibits the movement of dislocations, improving stability and promoting the formation of a thermodynamically nonequilibrium state, which can decrease the energy barrier for the adsorption, activation, and conversion of molecules [62,64,65,68]. Therefore, lattice distortion in HEAs reduces the overall system energy and thus enhances the activation and transport of active species. It may also be beneficial in oxidized materials for oxygen electrodes, where the formation of oxygen vacancies in high entropy oxides improves the catalytic performance for various oxidation processes [62]. Lattice strain (compressive or tensile) influences the interaction between metallic alloys and the adsorbate and can be tuned by selecting elements of different sizes. Additionally, the presence of various metallic elements on the surface, influenced by surface composition and microstructure, can create a redistribution of surface charges, resulting in alternating regions of accumulation and depletion. This tunability can lead to enhanced selectivity [66].

The confirmation of the favorable catalytic properties of high-entropy alloys can be found in the literature (see Table 1). Most of the recent publications about HEAs were focused on the correlation between fabrication and functional properties, along with their applications and upcoming challenges. Li et al., according to DFT calculations, found that Pt_18_Ni_26_Fe_15_Co_14_Cu_27_ high-entropy alloy with suitable electronic environment facilitates multi-active sites for proper adsorption of key intermediates and effective electron transfer during the electrocatalysis of methanol oxidation and hydrogen evolution reaction, enhancing the utilization of surface electroactivity [65]. Tong et al. explored (Fe,Co,Ni,Cu,Mo) quinary high-entropy alloy as a cathode material for SOEC. The (Fe,Co,Ni,Cu,Mo) HEA-based electrode layer offered boosted performance in the co-electrolysis process of H_2_O and CO_2_ for syngas production. An assembled SOEC utilizing the specially engineered quinary high-entropy alloy showed a notable rise in CO_2_ conversion capacity and greatly improved oxidation resistance compared to conventional Ni-based cathodes. The current density in electrolysis process increased by 18% (compared to nickel-based electrode), and a stability test lasting over 110 h showed no degradation. The same (Fe,Co,Ni,Cu,Mo) quinary high-entropy alloy with gadolinium-doped cerium dioxide (CGO) was also tested as an anode material in fuel cell mode and achieved a maximum power density of 0.48 W/cm^2^, whereas the Ni/CGO cell reached a peak power density of 0.39 W/cm^2^, indicating a performance improvement of over 20% at 850 °C. The difference between the two cell layers was also noticed in their polarization resistance (Rp): for the Ni-based cell, Rp was 0.88 Ω·cm^2^, while for the cell with the HEA electrode, it was reduced to about 0.66 Ω·cm^2^ [64]. Chen et al. considered FeCoNiCuX (X = Al, Mo) HEA with Ce_0.8_Sm_0.2_O_2_ (SDC) electrolyte as a potential intermediate temperature SOFC anode. FeCoNiCuX (X = Al, Mo)-SDC anode in fuel cell mode showed similar high conductivity with a Ni-based fuel electrode and exhibited exceptional catalytic activity for H_2_, CH_4_, and CO_2_. The LSGM electrolyte-supported cell with FeCoNiCuAl-SDC anode reached peak power densities of 779 and 526 mW·cm^−2^ when powered by H_2_ and CH_4_ at 850 °C, respectively. An XPS technique allowed for the conclusion that FeCoNiCuX (X = Al, Mo) HEAs with metals in different valence states provided more catalytically active sites, which enhanced activity in H_2_, CH_4_, and CO_2_ catalysis [69]. K. X. Lee et. al. found that GDC electrolyte with NiCoCuFeMn high-entropy alloy showed promising properties as an anode material for the internal utilization of methane in SOFC. Owing to the optimized mixture of HEA constituents, the NiCoCuFeMn/GDC composite exhibited a moderate reforming rate and outstanding resistance to carbon deposition under CH_4_ reforming conditions. The cell operated for 30 h without any signs of degradation. The polarization, including both ohmic and electrode resistances, remained low and stable. NiCoCuFeMn/GDC also showed flexibility for the fuel. The working stability for CH_4_ conversion over 30 h and the post-mortem analysis of the catalyst confirmed excellent carbon poisoning resistance, while both Ni/YSZ and Ni/GDC electrodes showed deactivation during cell working, regardless of their high initial CH_4_ conversion and H_2_ yield [70].

Efforts have also been made to exsolve multicomponent alloys *in situ*, directly from perovskite oxide. Shah et al. found that the highly dispersed NiFeCoCuPd nanoparticles can be exsolved from LaFeO_3_ perovskite and exhibit a lower catalytic activity but excellent working stability compared to exsolved NiFe and NiFeCo alloys for the dry reforming methane (DRM) reaction, with a minimal decline of CH_4_ and a CO_2_ conversion of ~2% during 24 h on-stream. It confirms that utilizing multiple metals to produce complex concentrated alloys can yield superior stability, selectivity, and activity compared to conventional binary or ternary alloys [48]. Based on the literature reports, we can state that HEAs as anode materials can improve electrode material properties for the direct utilization of not only pure hydrogen, but also hydrocarbons. The catalytic applications of selected high- and medium-entropy alloys are summarized in Table 1.

Recent investigations into the catalytic properties of multicomponent alloys clearly indicate that their notable catalytic activity, outstanding selectivity, and thermodynamic stability make high-entropy alloys the most promising materials for the upcoming future. HEAs can also be viewed positively from a financial cost perspective. Currently, precious metals like Au, Ag, Ru, Rh, Pd, and Pt are considered highly catalytically active elements for various reactions to enhance the process efficiency [66]. For instance, Pd can effectively promote the water splitting process and facilitate hydrogen evolution reaction under alkaline conditions [68]. However, noble metals are precious and expensive, so it is crucial to minimize the demand for precious metals in catalysts and improve their activity and stability. In HEAs, different metals can tune the catalytic properties (such as absorption energy) of the alloys. This approach includes adding low-cost materials as a diluent and presents an innovative solution to reduce the cost of high-priced catalysts [48,66].

The originality of the concept of obtaining electrode materials with high entropy in situ exsolved nanocatalysts for SOCs highlights the lack of literature reports on this topic. From over 500 articles published from 2013 to 2023, the majority (~60%) of the literature concerns monometallic in situ exsolution catalysts for electrochemical and thermochemical reactions (see Figure 5b) [48]. 

**Table 1:** Medium- and high-entropy alloys for thermocatalysis and electrocatalysis.

Catalyst	Crystal Structure	Synthesis Method	Material’s Catalytic Function	Reference
PdFeCoNiCu	Body-centered cubic	Colloidal synthesis (oil phase synthesis)	Hydrogen evolution reaction	[68]
NiFeCoCuPd (exsolved from LaFe_0.7_Ni_0.1_Co_0.1_Cu_0.05_Pd_0.05_O_3_ in reducing atmosphere)	Inhomogeneous metallic phase	Soft chemistry method (similar to the citric acid sol–gel-type Pechini method)	Catalytic dry methane reforming	[48,74]
Pt_18_Ni_26_Fe_15_Co_14_Cu_27_	Face-centered cubic	One-pot oil phase synthesis	Hydrogen evolution reaction; methanol oxidation reaction	[65]
NiCoCuFeMn	Crystal metal alloys	Co-precipitation method	Steam reforming of methane	[70]
Fe_0.1_Co_0.35_Ni_0.35_Cu_0.1_Mo_0.1_	Face-centered cubic	Reducing the corresponding oxide powder synthesized by sol-gel method	Cathode material in SOEC to co-electrolysis of H_2_O and CO_2_ toward syngas; anode material in SOFC	[64]
FeCoNiCuX (X = Al, Mo)	Face-centered cubic and Body-centered cubic	Mechanical alloying method in Ar atmosphere	SOFC anode, fueled by H_2_, CH_4_, and CO_2_	[69]
Ni_9.75_Co_13_Cu_16.25_Fe_16.25_Mn_9.75_	Face-centered cubic	Co-precipitation method	SOFC for steam reforming of methane	[70]

The concept of developing materials capable of the in situ exsolution of five or more metals to form high-entropy nanocatalysts on the surface is undoubtedly novel and innovative. Utilizing high-entropy alloys in catalysis is a relatively new area of research. Many factors govern catalysis, such as large surface area or optimum adsorption energetics. High-entropy alloys can meet all requirements, making them highly effective for many catalytic reactions. Some strategically designed HEAs exhibit outstanding resistance to carbon poisoning in SOFCs; however, their working mechanism has not yet been studied in detail [23]. Knowing the potential benefits, efforts should be made to obtain high-entropy exsolved alloys with a fully or partially ordered structure with all the benefits it can provide. Shah et al. postulate that the phase of exsolved nanoparticles correlates with the reduction process temperature and dwell time [74]. Cui et al. indicated that the phase of the nanoparticles depends on their size. The phase of nanoparticles below 5 nm is partially or fully ordered, while those larger than 60 nm exhibit disorder or phase-separated heterostructures (Figure 5c) [72,74]. In light of this, obtaining the small exsolved nanoparticles (~10 nm) is desired to achieve fully ordered high entropy nanoparticles. Preserving the multicomponent nature of metallic alloys is most effectively achieved by maintaining the nanometric size of the nanocatalysts [73]. The fabrication of uniform, nanostructured HEAs usually requires advanced and expensive synthesis procedures and techniques such as Pulsing Liquid Alloys, where this phenomenon is induced by applying a polarizing voltage signal to the liquid alloy [75]. Moreover, these methods often require specific conditions, such as high temperature or specific pressure, and can lead to undesirable large particles and irregular morphology [68].

### 4.3. Nanofiber Electrodes for Solid Oxide Cells

**Figure 6:**
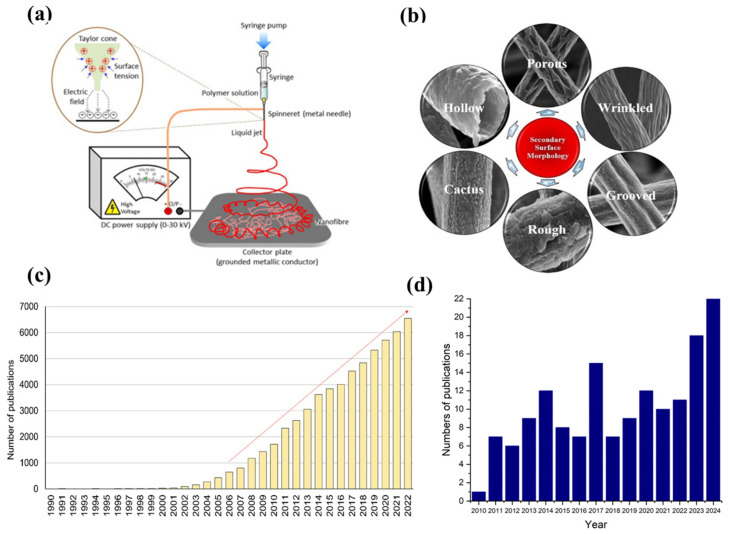
(**a**) The schematic of electrospinning process. Reprinted with permission from [82]. Copyright 2025, Elsevier. (**b**) Variability of nanofiber morphology depending on synthesis conditions. Reprinted with permission from [81]. Copyright 2020, Wiley. (**c**) Progress in electrospinning research based on publication output over the years. Reprinted with permission from [83]. Copyright 2023, Wiley. (**d**) Number of publications on nanofibers for SOCs application (based on Web of Science).

Morphology modification is another strategy that can noticeably improve electrodes performance. Such modification can be achieved by employing an electrospinning technique to produce materials with nanofiber morphology. The technique uses a high-voltage electric field to draw a charged polymer solution or melt it into very thin fibers. During the transit of the charged fluid from a needle (or spinneret) to a grounded collector, solvent evaporation induces the polymer to solidify, thereby yielding continuous fibers (Figure 6a). The interest in applying this approach is due to its simplicity, cost-effectiveness, and possibilities for producing one-dimensional (1D) nanostructures [76]. Attributed to the high specific surface area, a large number of reactive sites, robust connectivity, high porosity promoting rapid gas diffusion, and continuous charge transport pathways, nanofiber-structured materials have emerged as promising high-performance electrodes. These features not only boost electrode reactions, but also allow flexible operation in different modes, significantly increasing the performance of r-SOCs [29,76,77,78,79,80]. The continuous fibrous structure may also enhance cation diffusion, as evidenced by the increased nanoparticle population observed on thin fiber surface [45]. The combination of these features could overcome some of the inherent limitations associated with conventional electrode materials. From a technical standpoint, the electrospinning process may appear relatively straightforward; however, numerous factors influence fiber morphology. These factors may be broadly divided into three primary categories: (1) *solution properties*, which include surface tension, polymer concentration, molecular weight, viscosity, and solution conductivity; (2) *processing conditions*, comprising applied voltage, flow rate, needle diameter, and the collector distance from the spinneret tip; and (3) *environmental factors*, such as temperature and humidity, whose effects have been thoroughly discussed in the literature [79]. Moreover, the sintering process represents an additional key parameter; as shown in Figure 6b, the ultimate morphology of the nanofibers is profoundly influenced by the overall process parameters [81]. Figure 6c shows the annual number of publications on electrospinning, illustrating that interest in this technique began to surge in the 21st century. Meanwhile, Figure 6d illustrates the annual number of publications over the past 15 years (based on Web of Science), in which researchers used the electrospinning technique to fabricate components for SOCs. As the figure shows, the publication trend has remained relatively stable over the years; however, over the past two years, the number of published articles has experienced a marked increase. Owing to the versatility of this technique, researchers have utilized it to synthesize both anodes and cathodes with diverse architectures (e.g., core–shell, self-assembled multiphase, the inclusion of in situ exsolved nanoparticles) for applications in both SOFC and SOEC. 

For instance, Gumeci et al. electrospun a PrBa_0.5_Sr_0.5_Co_1.5_Fe_0.5_O_5+δ_ cathode material, which exhibits an Rp of 0.025 Ω·cm^2^ and a peak power density (PPD) of 2.5 W·cm^−2^ at 750 °C [84]. Chen et al. found that nanofibers exhibit significant potential as highly active air electrodes for reversible SOCs. A single PrBa_0.8_Ca_0.2_Co_2_O_5+δ_ nanofiber-based SOC exhibited great PPD (1.97 W·cm^−2^) and electrolysis performance (1.3 A·cm^−2^ at 1.3 V), and maintained operational stability at 750 °C over a period of 200 h [85]. The synthesis of a cobalt-free Sr_2_Fe_1.5_Mo_0.5_O_6−δ_ cathode for a symmetrical SOEC in nanofiber form reduced the Rp from 2.01 to 1.61 Ω·cm^2^ and increased the current density from 0.732 to 1.173 A·cm^−2^ under 2.0 V at 800 °C in a CO:CO_2_ (2:1) atmosphere compared to the corresponding powder material [86]. Furthermore, a symmetrical cell containing a GDC electrolyte and Sm_0.5_Sr_0.5_CoO_3−δ_ (SSC) nanofiber electrodes demonstrated an Rp of 0.010 Ω·cm^2^ at 700 °C and a PPD of 1.09 W·cm^−2^, both lower than those observed for the powder counterpart [87]. As mentioned above, electrospinning is a highly versatile technique, and therefore the production of more complex materials poses no significant challenge. In 2012, Zhi et al. reported a nanofibrous La_0.6_Sr_0.4_Co_0.2_Fe_0.8_O_3−δ_ (LSCF) cathode with a PPD of 0.9 W·cm^−2^ at 750 °C; its performance was subsequently enhanced to 1.07 W·cm^−2^ at the same temperature by infiltrating 20 wt% GDC into the material [88]. Since then, numerous studies have further enhanced the performance of LSCF-based nanofibers in various ways [89,90,91,92,93,94,95,96,97]. For instance, Zhang et al. showed that LSCF–CeO_2_ nanofibrous composites fabricated via electrospinning exhibited an Rp of 0.154 Ω·cm^2^ at 600 °C, significantly lower than that of LSCF powder (0.961 Ω·cm^2^) or LSCF nanofibers (0.537 Ω·cm^2^) [90]. Moreover, similar La_0.6_Sr_0.4_Co_0.2_Fe_0.8_O_3₋δ_@PrO_2−δ_ core–shell nanofibers exhibited an Rp of 0.076 Ω·cm^2^ at 700 °C, representing a 35% and 60% reduction compared to pure LSCF nanofibers (0.117 Ω·cm^2^) and LSCF powder (0.192 Ω·cm^2^), respectively [89]. A La_0.75_Sr_0.25_Cr_0.5_Mn_0.5_O_3_@Sm_0.2_Ce_0.8_O_1.9_ (LSCM@SDC) composite anode, attained via a single-nozzle electrospinning method for hydrocarbon fuel applications, exhibited an Rp of 0.76 Ω·cm^2^ in wet CH_4_ at 700 °C [29]. Similarly, the Ba_0.9_La_0.1_Co_0.7_Fe_0.2_Nb_0.1_O_3−δ_–Sm_0.2_Ce_0.8_O_2−δ_ (BLCFN-SDC) composite fibers further prove that morphological modifications significantly enhance performance: the BLCFN-SDC fibers exhibited an Rp of 0.05 Ω·cm^2^ at 700 °C, compared to 0.73 Ω·cm^2^ for BLCFN powder and 0.68 Ω·cm^2^ for BLCFN fibers [98].

### 4.4. Nanofibers with In Situ Exsolved Nanoparticles

**Figure 7:**
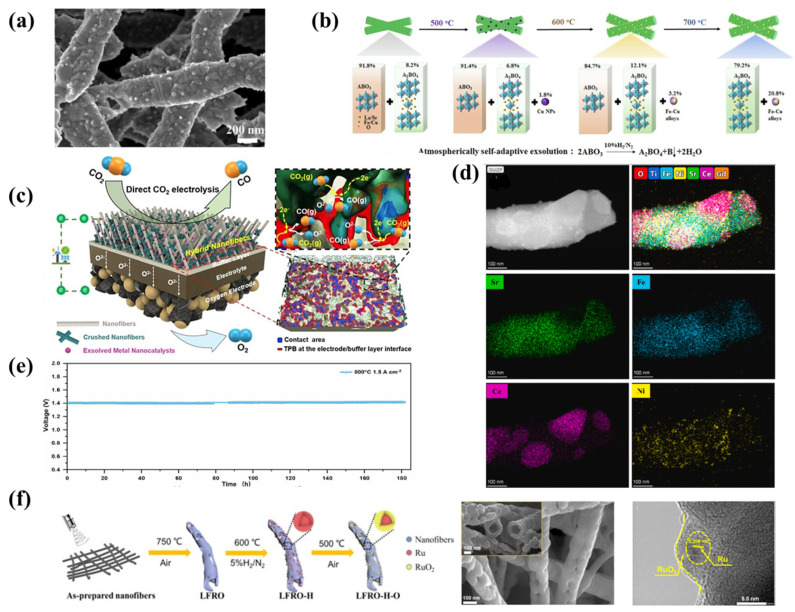
(**a**) SEM images of Ag-PBSC. Reprinted with permission from [101]. Copyright 2022, Elsevier. (**b**) Composition evolution under the reducing atmosphere with the different applied temperatures. Reprinted with permission from [103]. Copyright 2024, Elsevier. (**c**) Schematic illustration of H-LSCFP cell. Reprinted with permission from [105]. Copyright 2024, Springer Nature. (**d**) High-angle annular dark-field STEM image and EDX analysis of Ni@STFN/GDCN nanofibrous composite. Reprinted with permission from [106]. Copyright 2025, Elsevier. (**e**) Long-term stability of Ni@STFN/GDCN. Reprinted with permission from [106]. Copyright 2025, Elsevier. (**f**) Schematic illustration of La_0.9_Fe_0.92_Ru_0.08_O_3−δ_ synthesis and TEM analysis. Reprinted with permission from [107]. Copyright 2021, Elsevier.

As mentioned in previous chapters, appropriate material doping and the application of supplementary techniques can promote the in situ exsolution of nanoparticles on the surface, particularly under reducing conditions. It confirms that the morphology control with the use of electrospinning may be beneficial for the exsolution process because it allows for the formation of nanoscale particles ranging from a few to several dozen nanometers with high areal density. The efficiency of SOCs can be significantly increased by using nanofibrous electrodes with in situ exsolved nanocatalysts [29]. To date, most research on this phenomenon has predominantly focused on powder-based materials; however, it stands to reason that nanofibrous materials could similarly facilitate nanoparticle exsolution. Indeed, recent studies have reported in situ exsolution phenomena occurring in nanofibrous structures. For instance, Xu et al. developed an in situ technique to creating finely dispersed Ni-decorated Pt nanoparticles (1–6 nm) (Figure 5d) on perovskite La_0.52_Ca_0.28_Ni_0.06_Ti_0.94_O_3_ nanofibers with 0.2 deficiency on A-site. The deposition of Pt (0.5 wt%), via metal–support interaction, enhanced the reducibility and promoted Ni nucleation, resulting in nanometric alloy particles. The stable nanofiber structure covered with nanocatalysts demonstrated good catalytic performance for CO conversion, owing to the advantageous interplay between the metal and its support, as well as increased surface area which tended to reduce exsolved particle size [73]. Fang et al. investigated the catalytic properties of soot oxidation in PrBa_0.5_Sr_0.5_Co_0.5_Fe_1.8_O_5+δ_, a double perovskite structure material doped with cobalt in the B-site. Successfully fabricated porous nanotubes after reduction in a 10% H_2_/N_2_ atmosphere were covered by in situ exsolved Co/CoO_x_ core–shell nanoparticles. Their results demonstrated that the nanotubular material possesses significantly more macropores compared to bulk-type catalysts, which facilitate soot molecule transport. The presence of nanoparticulate catalysts enhances the catalytic activity and lowers the activation energy [99]. Zhou et al. prepared nanofibrous La_0.3_Sr_0.6_Ni_0.1_Ti_0.9_O_3_ (LSNT) material for r-SOCs. Under reducing conditions, nanoparticles measuring approximately 30 nm in diameter were formed on the surface. It was observed that most nanoparticles exsolved around pores, indicating that exsolution preferentially occurs in proximity of morphological defects or grain boundaries. The polarization resistance (Rp) of nanofibrous LSNT mixed with the GDC full cell was 0.27 Ω·cm^2^ at 800 °C in a 5% H_2_/N_2_ atmosphere and achieves a peak power density of 1.16 W cm^2^ at 800 °C, indicating better performance than powder LSNT electrode [100]. In another study, Pr_0.9_Ag_0.1_Ba_0.5_Sr_0.5_Co_2_O_5+δ_ (Ag-PBSC) nanofibrous material was synthesized for SOCs, demonstrating an alternative approach by doping silver into the A-site. After a 2-h reduction at 310 °C in a 5% H_2_/Ar atmosphere, nanoparticles with an average diameter of approximately 20 nm were exsolved on the surface (Figure 7a). A symmetrical cell fabricated from this material achieved a PPD of 0.704 W·cm^−2^ at 750 °C in fuel cell mode, while under an applied voltage of 1.5 V in electrolysis mode (50 vol% absolute humidity), a current density achieved 1.264 A·cm^−2^ at 750 °C [101]. Huang et al. synthesized La_0.5_Sr_0.6_Fe_0.8_Cu_0.15_Nb_0.05_O_3_ (LSFCN) nanofibers and investigated the exsolution process at various temperatures, as well as its influence on the oxygen evolution reaction (OER). Notably, the A-site composition reveals “overstoichiometry”, intended to yield a self-assembled, dual-phase material that comprises both a single perovskite and a Ruddlesden–Popper layered perovskite phase (Figure 7b). Although this approach for LSFCN was proposed by Song et al., their work focused on powder-based samples [102]. Finally, the research demonstrated that the best OER performance in alkaline conditions, featuring an overpotential of 381 mV at a current density of 10 mA·cm^−2^, was achieved using nanofibers reduced at 600 °C [103]. Xu et al. investigated La_0.52_Ca_0.28_Ni_0.06_Ti_0.94_O_3_ (LCNT), an A-site deficient perovskite with nickel in the B-site, using both solid-state reaction and electrospinning techniques to compare the results and better investigate the exsolution process. In general, they found that the average size of the nanoparticles exsolved from powder was smaller (15.9 nm) than those exsolved from nanofiber morphology (25.7 nm). Furthermore, the cell prepared using fibers exhibited a PPD of 0.380 W·cm^−2^ at 900 °C in hydrogen, which was slightly superior to that of the powder-based material. Additionally, they demonstrated that the perovskite fibers mass loss under hydrogen reduction indicates a faster exsolution process compared to the powder, suggesting the formation of a greater number of oxygen vacancies. Moreover, the Rp of the cell could be further enhanced through anodic polarization (“switching”) upon application of a voltage: the Rp decreased from 6.02 to 1.10 Ω·cm^2^ after switching at 1.8 V for 3 min and was further reduced to 0.69 Ω·cm^2^ after switching at 2.1 V [104]. Akhmadjonov et al. adopted a highly innovative approach by developing a hybrid La_0.6_Sr_0.4_Co_0.15_Fe_0.8_Pd_0.05_O_3−δ_ (H-LSCFP) material, in which low-aspect-ratio crushed LSCFP nanofibers were integrated into the excess porous interspaces of a high-aspect-ratio LSCFP nanofiber framework synthesized via electrospinning (Figure 7c). After reduction in pure H_2_, and CO_2_, respectively at 700 °C, cobalt and palladium were exsolved in situ on the surface. Furthermore, a quantitative 3D reconstruction analysis (used to create a digital twin of the nanofiber-structured electrodes) revealed that, compared to an LSCFP electrode without the crushed nanofibers (F-LSCFP), the H-LSCFP electrode exhibited a 190% increase in normalized contact area and a 147% increase in triple phase boundary per unit area. As a result, the SOEC equipped with the H-LSCFP electrode demonstrated a remarkable two-fold increase in current density (2.2 A cm^−2^) compared to the F-LSCFP cell (1.09 A cm^−2^) in 100% CO_2_ at 800 °C and 1.5 V [105]. Interesting results were presented by Wang et al., who employed electrospinning to fabricate a self-assembled nickel-doped Sr_0.95_Ti_0.3_Fe_0.7_O_3−δ_/Ce_0.9_Gd_0.05_Ni_0.05_O_2−δ_ (Ni@STFN/GDCN) composite material as a cathode for direct CO_2_ electrolysis in SOECs. Upon examining the reduced material in 10% H_2_/Ar using transmission electron microscopy (TEM), they observed a distinct segregation into two phases (Figure 7d), with exsolved nickel nanoparticles on the surface as small as 10 nm in diameter. Furthermore, when operated in pure CO_2_ at an applied voltage of 1.6 V, the cathode achieved a current density of 1.85 A·cm^−2^ and exhibited no degradation over 180 h of testing (Figure 7e) [106]. Fu et al. demonstrated a highly innovative method to improve the durability and OER performance of LaFeO_3_, a catalyst recognized for its potential in various applications, through strategic doping and morphological modifications. In their study, nonstoichiometry was introduced into the A-site and Ru was doped into the B-site, resulting in the synthesis of La_0.9_Fe_0.92_Ru_0.08_O_3−δ_ hollow fibers. Initially, the fibers were reduced in a 5% H_2_/N_2_ atmosphere to induce Ru in situ exsolution and subsequently oxidized at 500 °C for 1 h to form a core–shell Ru/RuO_2_ structure on the surface (Figure 7f), facilitating the OER kinetics [107]. In another study, the authors compared La_0.8_Ba_0.1_Mn_0.8_Ni_0.1_Cu_0.1_O_3_ synthesized in the form of nanofibers and as a sol–gel powder, evaluating its performance as a catalyst for the external steam reforming of liquid alcohols. To induce the in situ exsolution of the Ni nanoparticles, both forms underwent a reduction in a gas mixture containing 10% H_2_ in N_2_ at 600 °C for 3 h. The authors showed that the electrospinning technique considerably elevated the catalysts’ specific surface area and improved their catalytic performance, achieving nearly 100% methanol and ethanol conversion—outperforming the sol–gel powder material [108]. X. Xie investigated a nanofibrous Sr_0.95_Ti_0.3_Fe_0.7−x_Ni_x_O_3−δ_ with in situ exsolved nanoparticles for catalytic CO_2_ methanation. In their study, the authors optimized both the nickel content in the B-site and the sintering temperature, demonstrating that the optimal nickel content is x = 0.15 and the optimal sintering temperature is 1075 °C. Interestingly, at 1100 °C, the nanofibers began to degrade. After the reduction in pure hydrogen at 700 °C for one hour, additional Ni and Ni_3_Fe phases were observed, depending on the sintering temperature. Furthermore, the authors noted that higher calcination temperatures resulted in more nanoparticle formation due to enhanced nickel dissolution in the perovskite structure. At 450 °C, they achieved a CO_2_ conversion of 70.5% and a CH_4_ selectivity of 94.4% [109]. C. Yin et al. synthesized a La_0.3_Sr_0.6_Ti_0.1_Co_0.2_Fe_0.7_O_3−δ_ material for R-SOCs. Under reducing conditions, they observed the exsolution of a Co_3_Fe_7_ alloy on the surface. They also demonstrated that, during cycling, the fiber diameter gradually increased along with its specific surface area, while the exsolved nanoparticles became progressively smaller with each cycle [110].

## 5. Conclusions and Perspectives

The solid oxide cell technology is still under development and improvement. To enhance the overall performance of SOCs, various fuel electrode design strategies have been introduced, aimed at significantly improving the electrochemical properties of the cells. These strategies include utilizing the in situ exsolution of nanoparticles on the surface of matrix perovskites and developing nanofiber electrodes, contributing to the potential development of highly active catalysts for solid oxide cells. The in situ exsolution process, which partially embeds exsolved nanoparticles within the matrix, leads to the creation of fuel electrodes that are both catalytically active and stable. It is evident that increasing research attention is being directed towards the development of novel materials enhanced with exsolved nanoparticles for SOCs. While the in situ exsolution/dissolution mechanisms of nanoparticles still remain key areas of research, the efficient control of the morphology of exsolved nanoparticles and understanding their impact on performance enhancement require more systematic research and a deeper understanding. Furthermore, given the limited data available on the enduring stability of exsolved nanoparticles and perovskite-based heterostructured electrodes, comprehensive studies are still needed to ensure the long-term stable performance of these electrodes decorated with exsolved nanoparticles. Additionally, the exsolution of multi-elemental nanoparticles offers a promising approach to further enhance the stability and performance of electrodes, deserving greater attention from the scientific community. However, this area of research is still in its early stages. The crystal structure properties of exsolved multi-elemental nanoparticles need further investigation, and the potential formation of medium- or high-entropy nanoparticles is of significant scientific importance.

The use of nanofiber perovskite fuel electrodes has the potential to significantly enhance the performance of SOCs, thanks to their distinctive 3D porous nanofiber network structure, which offers high specific surface area, high porosity, excellent connectivity, and a continuous pathway for charge and mass transport. This structure also increases the number of reaction sites at the triple-phase boundaries. Interest in nanofiber electrodes has been steadily growing, with several promising findings reported in the literature. However, this area of research is still in its early stages, and several key challenges remain, such as understanding how different microstructures or morphologies of nanofibers (such as nanotubes, core–shell structured nanofibers) impact electrode performance and ensure their long-term stability. Nanofibers combined with in situ exsolved nanoparticles, which leverage the benefits of both the unique nanofiber microstructure and the catalytic activity and good stability of exsolved nanoparticles, hold great promise for enhancing fuel electrode performance and advancing high-performance SOC development. Nonetheless, research in this field is very limited, and more extensive studies are essential to further progress this field.

## Data Availability

Data are contained within the article.
